# Modulating mitochondrial metabolism: a neuroprotective mechanism for hypoxic–ischemic preconditioning

**DOI:** 10.1186/s13619-025-00268-4

**Published:** 2025-11-16

**Authors:** Wenxin Li, Guo Shao, Ruifang Qi

**Affiliations:** 1 Department of Basic and Forensic Medicine, Baotou Medical College, Baotou, People’s Republic of China; 2 Inner Mongolia Key Laboratory of Hypoxic Translational Medicine, Baotou Medical College, Baotou, People’s Republic of China; 3 Beijing Key Laboratory of Hypoxic Conditioning Translational Medicine, Xuanwu Hospital, Capital Medical University, Beijing, People’s Republic of China; 4 Longgang Institute of Medical Imaging, Shantou University Medical College & the Third People’s Hospital of Longgang District, Shenzhen, People’s Republic of China

**Keywords:** Hypoxia, Ischemia, Hypoxic/ischemic preconditioning, Mitochondrial, Neuroprotection

## Abstract

Hypoxia–ischemia plays a role in the physiological and pathological processes of various diseases and presents a common challenge for humans under extreme environmental conditions. Neurons are particularly sensitive to hypoxia–ischemia, and prolonged exposure may lead to irreversible brain damage. The primary mechanisms underlying this damage include energy depletion, mitochondrial dysfunction, oxidative stress, inflammation, and apoptosis. Mitochondria serve as primary organelles for adenosine triphosphate (ATP) production, and mitochondrial dysfunction plays a crucial role in mediating hypoxic pathophysiological processes. Hypoxic–ischemic preconditioning (H/IPC) is an endogenous cellular protective mechanism that reduces the damage caused by lethal hypoxic stressors. In this review, we summarize the potential role of H/IPC and its protective effects on mitochondrial quality control and function. This perspective offers a new approach for treating diseases caused by hypoxia–ischemia.

## Background

Oxygen is essential for maintaining cellular activity and energy supply. In most species, an adequate oxygen supply is crucial for various organs in the body to perform normal physiological functions (Thomas and Ashcroft 2019). Despite comprising only 2% of body weight, the brain accounts for approximately 20%–25% of the body’s oxygen consumption (Cardanho-Ramos et al. 2020). The brain is the most sensitive organ to hypoxia–ischemia. Both hypoxia and ischemia can result in brain damage because of an inadequate oxygen supply.

Hypoxia or ischemia in the brain can lead to significant metabolic changes in neurons and nonneuronal cells, resulting in varying degrees of neuroanatomical damage. Cerebral hypoxia–ischemia is a primary risk factor for stroke, neonatal hypoxic encephalopathy, and various neurodegenerative diseases. Additionally, hypoxia occurs in individuals exposed to extremely low-oxygen environments, such as at high altitudes, in aviation, and during navigation. Mitochondria are key targets of hypoxic–ischemic injury. When hypoxia–ischemia occurs, oxidative phosphorylation is impaired, leading to insufficient adenosine triphosphate (ATP) production and an energy crisis. Owing to the high energy demands of the nervous system, neurons are particularly susceptible to mitochondrial dysfunction. Impaired mitochondria are unable to produce sufficient levels of ATP to meet the energy needs of neurons (Cheng et al. 2022; Umbrasas et al. 2021).

Energy deficit-induced neuronal death remains a critical clinical problem for which no pharmacological therapy is currently available. Although therapeutic hypothermia remains the standard treatment and has been shown to reduce mortality to some extent, its therapeutic efficacy is limited (Deng et al. 2025). Therefore, exploring new protective mechanisms for treating hypoxic–ischemic brain damage is imperative. It is well known that hypoxic–ischemic preconditioning (H/IPC) supports ATP supply and reduces ATP demand through endogenous protective mechanisms. Remote ischemic preconditioning has been evaluated as a preclinical intervention for stroke. Similarly, hypoxic preconditioning is effective at protecting neurons from ischemic/hypoxic injury. Elucidating energy- or mitochondrial metabolism-related protective mechanisms may therefore offer new clinical insights for treating hypoxic–ischemic brain damage by activating endogenous pathways rather than by relying on pharmacological agents.

H/IPC refers to transient sublethal hypoxia/ischemic stimulation administered prior to a more severe ischemic or hypoxic event, enhancing the body’s tolerance to subsequent injury (Rybnikova and Samoilov 2015; Vlasov 2012). IPC is defined as transient sublethal ischemic injury, which may mobilize protective mechanisms to improve neuronal damage following fatal ischemia (Murry et al. 1986). The activation of multiple kinase pathways that converge on mitochondria to mediate protection has been implicated in the mechanism of IPC (Abel 2021). IPC is induced by both oxygen and nutrient deprivation, whereas HPC is induced by oxygen deprivation alone. Both IPC and HPC require repeated short-term stimulation to activate endogenous protective mechanisms. Both approaches can simultaneously trigger multiple protective responses, promoting ATP synthesis, thereby reducing ROS release, alleviating Ca^2+^ overload, reducing mitochondrial-mediated apoptosis, promoting mitochondrial autophagy, and ultimately reducing cell death (Zhu et al. 2025). The protective effects of H/IPC on organs such as the brain and heart have been widely demonstrated in animal models and cultured cells (Murry et al. 1986; Schurr et al. 1986).

Some clinical studies have shown that H/IPC represents a potential therapeutic treatment for patients affected by hypoxia–ischemia. Meng et al. reported that upper-limb IPC prevents recurrent stroke in patients with intracranial arterial stenosis (Meng et al. 2012). Ding et al*.* reported that remote ischemic conditioning (RIC) ameliorates the sequelae of ischemic moyamoya disease (Ding et al. 2020). RIC reduces the incidence of new ischemic events after brain tumor surgery (Sales et al. 2017). These studies revealed that RIC is a promising therapeutic approach for patients with ischemic stroke. These protective mechanisms often involve the common activation of various endogenous pathways and molecular responses that help mitigate tissue damage (Li et al. 2023; Liu et al. 2021a).

The model of the HPC also presents diversity. Intermittent hypoxia (IH) is often considered an effective mechanism of HPC, and many studies have demonstrated its role in neuroprotection. Wan et al. reported that preconditioning with intermittent hypobaric hypoxia (IHH) attenuates stroke damage and modulates endocytosis in residual neurons (Wan et al. 2021). Short-term moderate IH improved tolerance to acute high-altitude exposure (4500 m) and protected against acute hypoxic injury induced by exposure to sustained normobaric hypoxia (Wang et al. 2024). However, sustainable intermittent hypoxia is a major risk factor for obstructive sleep apnea syndrome (OSAS) (Zolotoff et al. 2021). Therefore, IH has both beneficial and detrimental effects, but H/IPC is a feasible, safe, and noninvasive treatment. Whether H/IPC exerts its neuroprotective effect by safeguarding mitochondria has garnered significant attention.

## H/IPC and mitochondrial quality control

**Fig.1:**
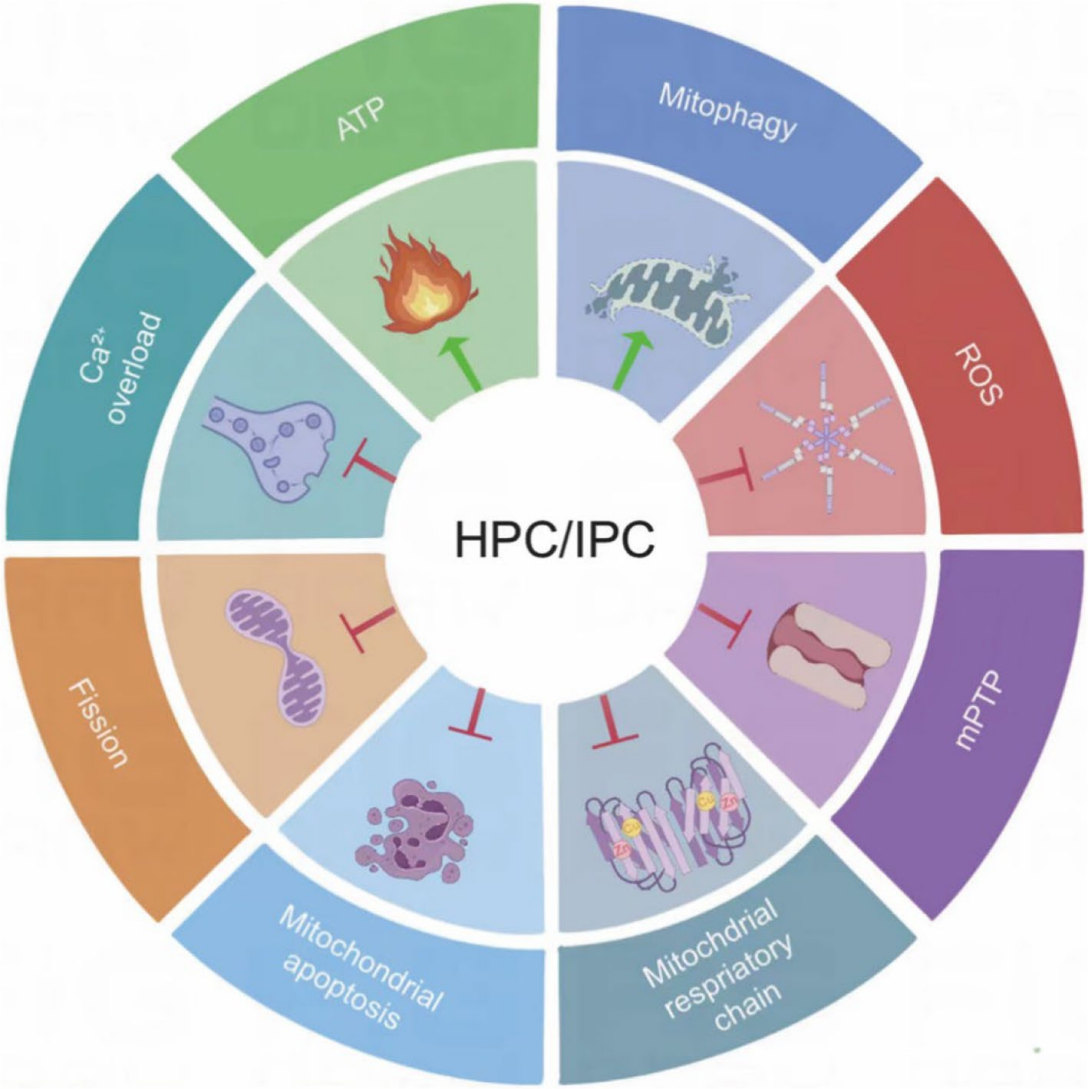
H/IPC regulated mitochondrial metabolism. Abbreviations: H/IPC, Hypoxic-ischemic preconditioning; ATP, adenosine triphosphate; Mitophagy, mitochondrial autophagy; ROS, reactive oxygen species; mPTP, mitochondrial permeability transition pore

Mitochondria are double-membrane organelles that contain their own DNA and replicate independently of host cells. They are mainly responsible for ATP production and the regulation of cellular energy metabolism. Most cells, especially nerve cells, possess abundant mitochondria to meet the constant high energy demand. Additionally, mitochondria play a central role in calcium (Ca^2+^) homeostasis, redox regulation, fatty acid synthesis, oxidation, and apoptosis (Mitchell 1961; Neupane et al. 2019; Nunnari and Suomalainen 2012) (Fig. 1).

The electron transport chain (ETC) and oxidative phosphorylation in the mitochondria are the first to be affected when cells experience hypoxic–ischemic stress. This disruption destroys the integrity of cellular respiration, leading to insufficient ATP production and an energy crisis. Besides, mitochondria would produce a large number of reactive oxygen species (ROS), which activate mitochondrial autophagy and disrupt Ca^2+^ transport. These factors contribute to irreversible changes in mitochondrial membrane permeability and cytoplasmic Ca^2+^ overload, ultimately leading to mitochondrial dysfunction and apoptosis. As the duration of ischemic/hypoxic conditions increases, mitochondrial function and structure sustain damage. Decreased mitochondrial membrane potential (MMP) and ATP depletion further impair cell function (Bai et al. 2020; Nguyen et al. 2019; Yang et al. 2016). Therefore, protecting mitochondrial function is crucial in ischemic stroke.

H/IPC acts as an endogenous cellular protective mechanism and regulates mitochondrial quality control, which is a crucial regulatory machinery responsible for managing the size, quantity, morphology, quality, and biological activity of mitochondria (Ren and Zhang 2018). When cellular hypoxic or ischemic stress induces mitochondrial injury, H/IPC mitigates the effects by preserving the original structure and composition of cells through mechanisms such as mitochondrial antioxidants, DNA repair, and protein folding and degradation. If these initial defense mechanisms fail, a broader quality control system comes into play. This system involves mitochondrial biogenesis, fusion, and fission and the removal of damaged mitochondria through mitophagy (Cheng et al. 2022; Murry et al. 1986). This review focuses on the possible mechanisms through which mitochondrial metabolism is regulated by H/IPC and explores potential targets of H/IPC for the prevention and treatment of hypoxic–ischemic encephalopathy or extremely hypoxic environments.

### H/IPC and ATP

Mitochondria are central to cellular energy production via oxidative phosphorylation and the regulation of intracellular signaling cascades (Evans and Holzbaur 2020). Mitochondria utilize the ETC to generate usable chemical energy from electron donors such as reduced nicotinamide adenine dinucleotide (NADH) through a series of oxidation/reduction reactions. In these reactions, molecules transfer electrons, facilitating transmembrane proton transport and establishing an electrochemical gradient that drives ATP synthesis. An electrochemical gradient, called the proton-motive force (PMF), which crosses the mitochondrial inner membrane, is generated by proton-pumping respiratory complexes of the electron transport chain, and the PMF decreases under pathological hypoxic conditions (Dingley et al. 2010; Shadel and Horvath 2015).

Stroke is a common pathology in which cells undergo hypoxia and rapid reoxygenation, leading to changes in PMF and compromised mitochondrial function. During ischemic stroke, mitochondrial dysfunction primarily manifests as ATP depletion. This is attributed to the loss of the MMP during ischemia, as mitochondrial ATPase rapidly hydrolyzes ATP, contributing to its depletion and damaged mitochondrial quality (Umbrasas et al. 2021). The absence of oxygen and glucose inhibits mitochondrial respiration and, consequently, ATP synthesis. This leads to energy depletion and, ultimately, necrotic cell death (Huang et al. 2021; Zeng et al. 2021).

Xiao et al*.* reported that HPC attenuates propofol neurotoxicity by reducing mitochondrial damage and increasing ATP levels in the hippocampus of neonatal rats (Xiao et al. 2020). We found that HPC increased ATP levels in HT22 cells, improving their tolerance to hypoxia (Qi et al. 2022). Han et al*.* reported that remote ischemic preconditioning (RIPC) ameliorates high-altitude hypoxia-induced brain edema by significantly rescuing the MMP and stimulating glucose metabolism in the tricarboxylic acid cycle to increase mitochondrial ATP production (Han et al. 2024). Interestingly, Berry et al*.* engineered a mitochondrion-targeted light-activated proton pump called mitochondria-ON (mtON) to selectively increase the PMF in *Caenorhabditis elegans*. They reported that HPC transiently activates mtON in a dose-dependent manner to increase the PMF, support ATP synthesis, and enhance resistance to hypoxia. However, mtON activation during HPC decreases the protective effects, suggesting that HPC is mediated by a reduction in the PMF. mtON affects AMP-activated protein kinase (AMPK) metabolic signaling (Berry et al. 2020). From this perspective, PMF is sensitive to low oxygen, which can lead to a decrease in PMF, however, this decrease does not affect ATP synthesis.

AMPK is a crucial cellular energy sensor that can be activated by increasing the AMP/ATP ratio when cellular energy levels decrease because of cellular stresses such as glucose deprivation, inhibition of mitochondrial oxidative phosphorylation, or exposure to toxic agents (Dengler 2020). AMPK promotes ATP production by increasing the activity or expression of proteins involved in catabolism while conserving ATP by switching off biosynthetic pathways (Hardie et al. 2012). Intermittent hypoxia (IH) is an effective method for H/IPC and increases ATP content. This increase in ATP content may activate AMPK–proliferator-activated receptor γ coactivator-1α (PGC-1α)–silenced regulatory protein 3 (Sirt3) signaling, which repairs mitochondrial ultrastructural damage, thus improving mitochondrial function (Su et al. 2022).

Srtuins (Sirts) are a protein family with versatile functions, such as controlling mitochondrial metabolism (Ji et al. 2022). Sirtuin-1 (Sirt1) restores mitochondrial structure and function in rats by activating Sirt3 after cerebral ischemia/reperfusion injury (Chen et al. 2024). Sirt3 activates the mitochondrial unfolded protein response and reduces cerebral ischemia/reperfusion injury (Xiaowei et al. 2023). Yan et al*.* reported that Sirt3 mitigated neuroinflammation and mitochondrial damage after hypoxic–ischemic brain injury through increased ATP production (Yan et al. 2025). Hence, SIRT3 is considered a vital regulator of mitochondrial functions, antioxidative defense systems and energy metabolism (Liu et al. 2021c, 2020; Peng et al. 2020; Potthast et al. 2020).

**Fig.2:**
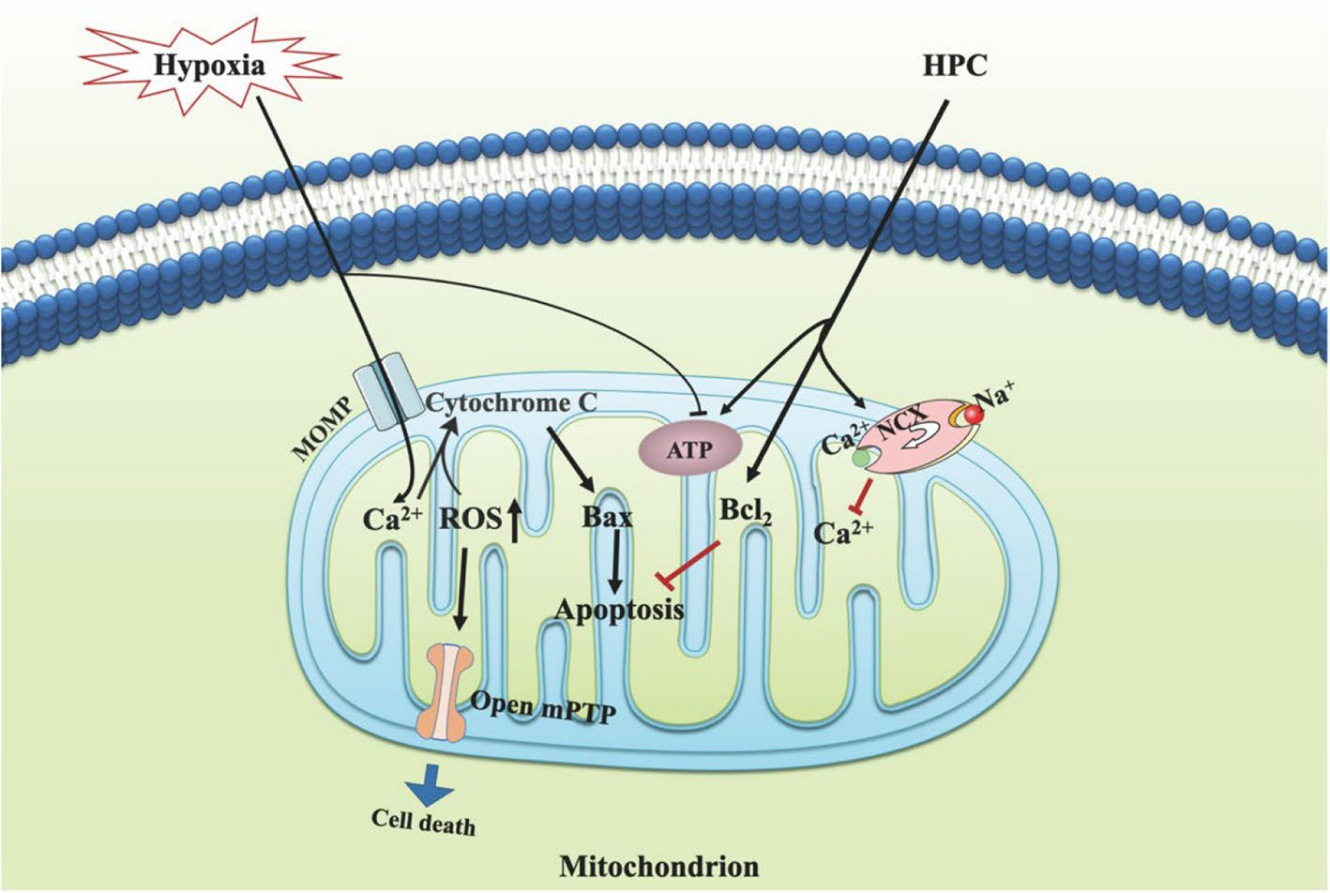
H/IPC and ATP, ROS, Ca^2+^ and mitochondrial apoptosis. H/IPC protected mitochondria by increasing ATP concentration, reducing Ca^2+^ overload and ROS, and alleviating mitochondrial-mediated intrinsic apoptosis caused by hypoxic-ischemic injury. Abbreviations: MOMP, mitochondrial outer membrane pore; Bcl_2_, B Cell Lymphoma 2; Bax, BCL_2_-associated X protein; NCX, Na^+^-Ca^2+^ exchanger

Although there have been some studies exploring the molecular mechanisms that promote ATP synthesis, it is still unclear and more research is needed to investigate (Fig. 2). In eukaryotic cells, ATP generation is generally viewed as the primary function of mitochondria under normoxic conditions. In contrast, ROS are regarded as the byproducts of respiration and are widely associated with dysfunction and disease (Strzyz 2022). How does H/IPC regulate the ROS?

### H/IPC and ROS

ROS refer to superoxide anions (O_2_^•^‾), hydrogen peroxide (H_2_O_2_), or their derivatives. Under physiological conditions, most ROS (approximately 95%) are generated in the ETC. ROS are important complex I metabolites that play key roles in the regulation of cellular metabolism (Okoye et al. 2023).

The ETC produces low levels of ROS in the form of O_2_^•^‾, which is rapidly converted to H_2_O_2_ through dismutation under normal metabolic activity. H_2_O_2_ functions as a secondary messenger, similar to Ca^2+^, as it readily crosses the membrane. Mitochondrial ROS (mtROS) act as metabolic signals that coordinate bioenergetic states, cellular activity, and adaptation. This basal redox tone is called oxidative eustress. Oxidative stress occurs when there is an imbalance between the production of reactive oxygen species (ROS) and the cell’s antioxidant defenses (D'Apolito et al. 2024).

According to Tan et al*.*, a moderate increase in ROS induces autophagy to protect SY5Y cells against oxidative stress (Tan et al. 2018). The findings of Xu et al*.* were similar to those of Tan et al*.*, who reported that atmospheric pressure plasma preconditioning activated autophagy by upregulating ROS and reducing SH-SY5Y apoptosis induced by oxygen–glucose deprivation (OGD) in vitro, an effect that was attenuated by the ROS scavenger N-acetyl-L-cysteine (Yan et al. 2024). However, pathological conditions such as severe hypoxia or ischemia increase ROS production in the mitochondria. Mitochondria are the major targets of ROS, and ROS accumulate and are aberrantly oxidized, which can damage cellular components such as lipids, proteins, and DNA, making them dysfunctional and ultimately inducing apoptosis (Koren et al. 2023; Venditti and Di Meo 2020). This damage to mitochondrial quality is considered oxidative distress.

Numerous studies have demonstrated that ROS levels are elevated by cerebral ischemia‒reperfusion injury (CIRI) (Wang et al. 2022; Zeng et al. 2022) and that H/IPC downregulates ROS levels to alleviate CIRI. Chen reported that HPC reverses the neurotoxic effects of propofol, leading to increased hippocampal neuron viability, increased MMP, increased superoxide dismutase content, reduced ROS levels, elevated Fe^2+^ levels, decreased neuronal apoptosis, altered expression of ferroptosis-related proteins, and inhibition of ferroptosis (Chen et al. 2023). HPC also increases astrocyte viability and promotes the secretion of neuroprotective factors. Additionally, HPC increases the expression of antioxidant enzymes and enhances mitochondrial function, thus reducing oxidative stress induced by OGD (Wu et al. 2021b). Ma et al*.* reported that IPC neurons protect astrocytes against ischemia/reperfusion (I/R)-induced injury by inhibiting oxidative stress, leading to reduced ROS levels (Ma et al. 2018). Wu et al*.* reported that HPC increases the glucose transport activity of neurons in brain-injured rats. It achieves this by upregulating hypoxia-inducible factor-1α (HIF-1α) and increasing the expression and activity of two glucose transporters, GLUT1 and GLUT3, in the brain, maintaining intracellular glucose levels and ensuring the energy supply of neurons, thus reducing neuronal injury (Wu et al. 2021a) (Fig. 2).

HIF-1α and HIF-2α are oxygen-sensitive transcription factors that mediate the adaptive metabolic response to hypoxia and play key roles in ischemia. HIF-1α protects against oxidative stress by directly targeting mitochondria, and the expression of the mitochondrial-targeted form of HIF-1α (mito-HIF-1α) reduces ROS production, oxidative stress-induced apoptosis and the collapse of the MMP (Li et al. 2019). Gardiner et al*.* reported that in retinopathy of prematurity (ROP), the expression of AMPK, PGC1α and HIF-2α was visible at the lens equator during secondary fiber differentiation and reflected changes in size, mitochondrial content, and metabolism (Gardiner et al. 2022).

These studies reveal that ROS have two functions. On the one hand, ROS may alleviate apoptosis by activating autophagy. On the other hand, excessive ROS levels can cause oxidative stress and increase apoptosis. Therefore, ROS activation is often considered a “double-edged sword”. Mitochondria produce ROS during metabolism, which play a further role in redox signaling, however, excessive ROS may also act as an additional trigger for abnormal mitochondrial Ca^2+^ handling, resulting in mitochondrial Ca^2+^ overload (Baev et al. 2022). H/IPC reduces excessive accumulation of intracellular ROS, thereby alleviating oxidative stress, how does H/IPC affect Ca^2+^ overload?

### H/IPC and Ca^2+^ overload

Neurons are high-energy-demanding cells that require tight regulation of Ca^2+^ to maintain their action potentials (Hees and Harbauer 2022). Ca^2+^ is a pleiotropic second messenger in cells that plays crucial roles in neuronal signal transduction, the regulation of neuronal excitability, and various cellular functions, such as gene transcription and cell proliferation (Hernansanz-Agustin et al. 2020). Mitochondria play a fundamental role in Ca^2+^ homeostasis because mitochondrial Ca^2+^ is a key regulator of oxidative metabolism.

Hypoxia-induced Ca^2+^ overload leads to increased mitochondrial oxidative stress. An increase in the intracellular free Ca^2+^ concentration leads to increased mitochondrial membrane depolarization. Mitochondrial Ca^2+^ overload triggers the opening of the mitochondrial permeability transition pore (mPTP), and mitochondrial quality is affected, which subsequently causes the release of caspase cofactors. These factors initiate the apoptotic process, ultimately leading to cell death (D'Angelo et al. 2023; Filadi and Greotti 2021).

Consequently, precise control of the Ca^2+^ concentration through the modulation of calcium channels is crucial for regulating both physiological and pathophysiological conditions (Akyuva and Naziroglu 2020). Mitochondrial Ca^2+^ uptake is mediated mainly by mitochondrial Ca^2+^ uniporters, whereas mitochondrial Ca^2+^ efflux is regulated by Na^+^-Ca2 + exchangers and H^+^/Ca^2+^ exchangers (Filadi and Greotti 2021). Hypoxic–ischemic insults induce a reduction in NCX1 and NCX3 expression, and enhancing NCX activity can alleviate hypoxic–ischemic damage (Cerullo et al. 2018).

Brancaccio reported that the HPC protects hypoxic–ischemic (HI) neonatal mice from NCX. HPC increases NCX1- and NCX3-positive cells in the dentate gyrus, contributing to the maintenance of ionic homeostasis (Brancaccio et al. 2022). IPC-mediated upregulation of NCX1 and NCX3 activity reduces mitochondrial Ca^2+^ concentrations (Sisalli et al. 2014). Additionally, HPC reduces the phosphorylation of mixed lineage kinase domain-like proteins in both neurons and microglia in the CA1 region following transient global cerebral ischemia, and it inhibits Ca^2+^ influx to counter Ca^2+^ overload (Liu et al. 2021b).

Mitochondria are important cell death checkpoints, and mitochondrial Ca^2+^ overload is considered a potent intrinsic apoptotic pathway inducer. The mechanism of mitochondrial pathway apoptosis may involve elevated levels of ROS and Ca^2+^ influx in hypoxia–ischemia, which lead to a membrane permeability transition and the release of cytochrome c from the mitochondrial intermembrane space (Marzetti et al. 2013). Therefore, H/IPC plays an important role in neuroprotection by regulating calcium balance to reduce apoptosis (Fig. 2).

### H/IPC and mitochondrial-mediated intrinsic apoptosis pathways

Apoptosis is an evolutionarily conserved programmed cell death pathway responsible for the effective elimination of cells during normal eukaryotic development and maintenance of organismal homeostasis. It is initiated by either internal or external stimuli and is mediated by two distinct pathways: the intrinsic pathway (mitochondria-mediated) and the extrinsic pathway (death receptor [G]-mediated). The regulation and execution of mitochondria-mediated proteins are governed by the B-cell lymphoma 2 (BCL-2) family of proteins, which includes both pro-apoptotic (such as Bax) and pro-survival (anti-apoptotic) members. Precise modulation of the balance between these two groups largely determines the choice between cell life and death (Singh et al. 2019) (Fig. 2).

Hypoxic–ischemic insults are associated with increased mitochondria-mediated apoptosis. Neonatal hypoxic–ischemic conditions increase mitochondrial swelling, the loss of the MMP, and the induction of oxidative stress. This triggers apoptosome activation and caspase-mediated apoptosis (Odorcyk et al. 2021). Neurons in the brain tissue of neonatal rats with hypoxic–ischemic brain damage exhibit a decreased number, disordered arrangement, and activation of the mitochondrial apoptotic pathway (Wang et al. 2021). Cerebral ischemia increases apoptosis, which destroys mitochondrial structural integrity, thereby impairing mitochondrial quality control (Wu et al. 2024).

Previous studies have revealed that IPC effectively mitigates brain ischemia-related damage. IPC can reduce the Bax/Bcl-2 ratio and decrease the activation of Caspase3, thereby alleviating ischemic brain injury (Sun et al. 2021). Lv et al*.* reported that RIPC also inhibits apoptosis through the intrinsic pathway, increases the formation of mitochondria-derived vesicles, and alleviates cerebral ischemia–reperfusion injury (Lv et al. 2020). RIPC provides protection against ischemic stroke by suppressing the mitochondrial-mediated apoptosis pathway (Wu et al. 2021b). HPC also affects the mitochondrial apoptosis pathway for neuroprotection (Huang et al. 2020). HPC mitigates neurological impairment induced by I/R injury, significantly reducing cerebral infarction volume, attenuating pathological damage to the hippocampus, decreasing apoptosis, and downregulating proinflammatory factors (Pang et al. 2022). Additionally, HPC promotes microglial polarization, enhances the expression of the antiapoptotic gene Bcl-2, and decreases the expression of the proapoptotic gene Bax to mitigate ischemic cerebral injury (Huang et al. 2019).

The molecular mechanisms involved in apoptosis through mitochondrial pathways vary. IPC protects astrocytes by inhibiting pro-apoptotic signals through erythropoietin (EPO), activating anti-apoptotic proteins via the phosphatidylinositol 3-kinase/extracellular signal-regulated kinase/signal transducer and activator of transcription 5 pathway, and stimulating the activation of antioxidant proteins by upregulating the activity of antioxidant enzymes (Wu et al. 2017). According to Liang et al*.*, RIPC preactivated the Notch1/NF-κB pathway, reduces Bax expression, increases Bcl-2 expression, and reduces neuronal apoptosis (Liang et al. 2019). Zhang et al*.* reported that HPC provided neuroprotective effects by increasing antioxidant activity and EPO expression and preventing apoptosis and astrogliosis in the brains of adult rats exposed to acute severe hypoxia (Murry et al. 1986). HPC also promotes microglial angiogenesis and inhibits apoptosis in stroke mice via the transforming growth factor/Smad2/3 pathway (Zhang et al. 2021b). Guan et al*.* suggested that HPC inhibits the intrinsic apoptotic pathway by upregulating cAMP and phosphorylating CREB, thus exerting neuroprotective effects (Guan et al. 2019).

These studies indicate that mitochondria-mediated intrinsic apoptosis is key to the neuroprotective effects of H/IPC. Our early research revealed that HPC increases the viability of HT22 cells and reduces apoptosis; however, further research is needed to determine whether these effects occur through the mitochondrial apoptosis pathway (Qi et al. 2022). To reduce damage to cells, the damaged mitochondrial fragments produced by apoptosis need to be cleared in a timely manner. Mitophagy is a form of autophagy that selectively removes damaged mitochondria and attenuates mitochondria-dependent apoptosis. In addition, it may also be among the targets of H/IPC.

### H/IPC and mitophagy

Autophagy is a conserved lysosomal degradation pathway that involves the engulfment of cargo for degradation by double-membrane organelles. Three main types of autophagy have been described—macroautophagy, microautophagy, and chaperone-mediated autophagy. Macroautophagy participates in the turnover of damaged organelles such as mitochondria (mitophagy), ribosomes (ribophagy), and endoplasmic reticulum (ER-phagy) (Zhang et al. 2021a).

**Fig.3:**
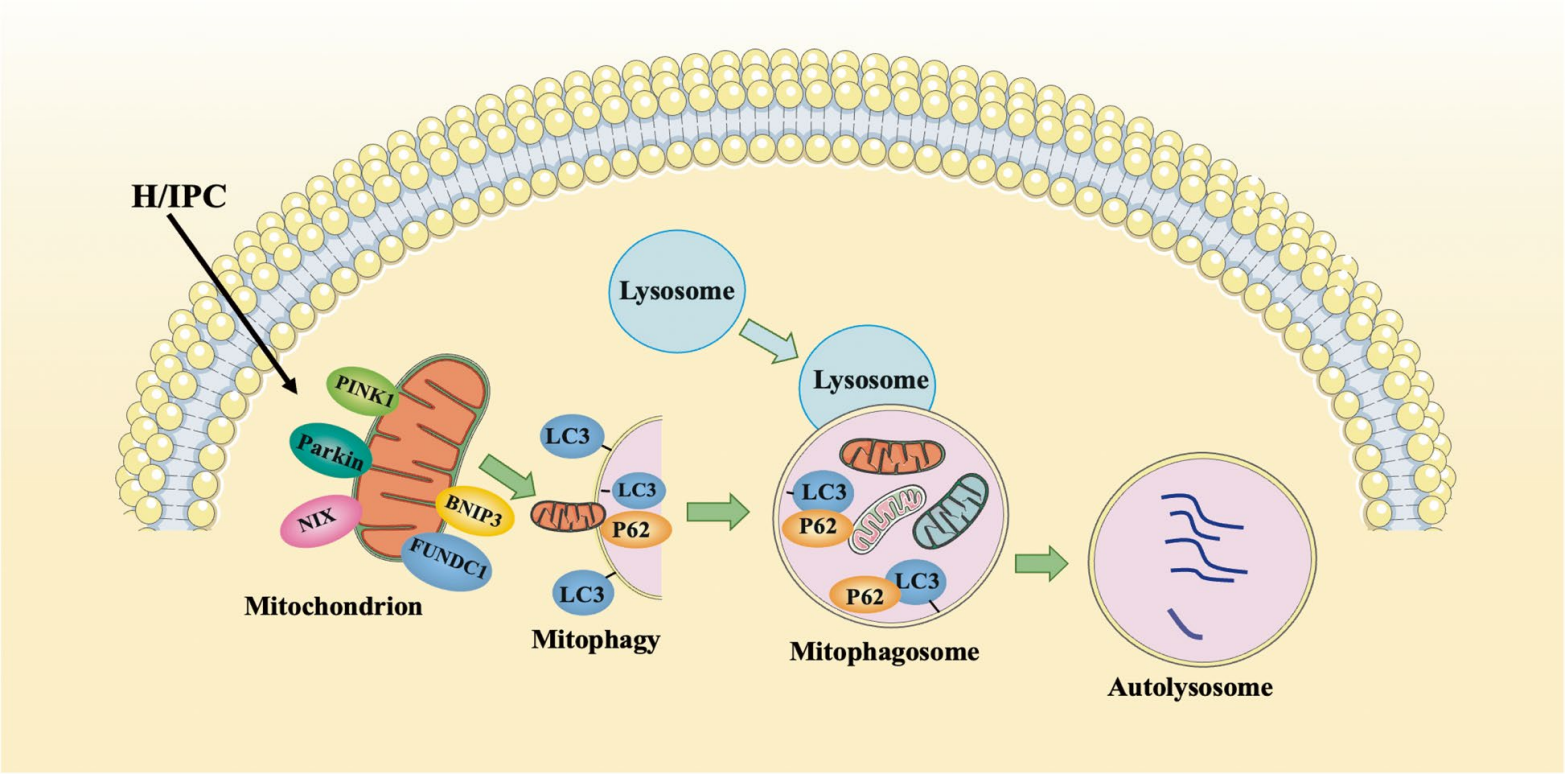
H/IPC promotes mitophagy. Abbreviations: PINK, phosphatase and tensin homolog (PTEN)-induced putative kinase 1; BNIP3, BCL-2 interacting protein 3; NIX, BNIP3-like (BNIP3L or NIP3-like protein, NIP3L; FUNDC1, FUN14 domain-containing 1; LC3, microtubule-associated protein 1 light chain 3; P62/SQSTM1, sequestosome-1

The mitochondrial network is a dynamically interconnected system of organelles that undergo continuous renewal and rearrangement to meet cellular requirements. Mitophagy is a specialized form of autophagy that selectively recognizes and removes damaged or superfluous mitochondria. It shares fundamental features and core proteins with general autophagy and is a crucial mechanism for cellular homeostasis (Chakrabarti and Higgs 2021; Zhang et al. 2022). There are currently two major types of mitophagy pathways: the ubiquitin-mediated pathway and the receptor-mediated pathway. The ubiquitin-mediated pathway involves the PTEN-induced putative kinase protein 1 (PINK1) and Parkin-mediated ubiquitination processes. The receptor-mediated pathway includes BCL2-interacting protein 3 (BNIP3), BNIP3-like (BNIP3L or NIP3-like protein, NIP3L or NIX), FUN14 domain-containing 1 (FUNDC1), and other mitophagy receptors (Shen et al. 2021) (Fig. 3).

Mitophagy can be induced by various pathological processes, such as hypoxia, inflammation, oxidant injury, and other damaging insults. It serves as a stress response mechanism that inhibits mitochondria-dependent cell death (Xiong et al. 2019). He et al*.* reported that PC12 cells undergo mitophagy to increase their stability in response to hypoxia (He et al. 2022). Mitophagy is induced by tissue-type plasminogen activators and inhibits apoptosis to protect neurons against CIRI (Cai et al. 2021).

Park reported that hypoxically preconditioned human placenta-derived mesenchymal stem cell-derived extracellular vesicles (HPPSC_EVs), with a diameter of 170–180 nm, stimulate ATP production and mitigate hypoxic injury in R28 retinal progenitor cells by activating the p62 mitophagy signaling pathway. HPPSC_EVs significantly increase antioxidant levels in vivo, leading to the repair of mitochondrial damage in neuronal tissues. These effects were sustainable (Park et al. 2022). HPC promotes mitophagy against transient global cerebral ischemia in adult rats through the PINK1/Parkin-induced mitochondrial ubiquitination pathway (Wen et al. 2021). Furthermore, HPC rejuvenates mesenchymal stem cells and enhances neuroprotection following intracerebral hemorrhage via miR-326-mediated autophagy (Liu et al. 2021b). Currently, research on whether H/IPC affects receptor-mediated mitophagy is limited, and further studies are warranted.

Currently, the protective effects of H/IPC on mitochondrial autophagy have been primarily studied in the context of the kidneys and heart, with limited research being conducted to determine whether the nervous system undergoes mitochondrial autophagy. In our previous studies, we reported that autophagy is involved in the protective effects of HPC in mouse hippocampal CA1 and CA3 neurons. However, mitochondrial autophagy during this process has not yet been investigated. The quality control of mitochondria involves not only timely clearance of damaged mitochondria through mitophagy but also the combined effects of mitochondrial biogenesis, fission and fusion. The protection of nerves by H/IPC is closely related to these mitochondrial processes.

### H/IPC and mitochondrial biogenesis, fission, and fusion

Mitochondria are not generated de novo in eukaryotic cells; instead, preexisting mitochondria are distributed between the two daughter cells during cell division. Mitochondrial mass and quality are tightly regulated by two essential and opposing mechanisms in response to cellular energy demands and other cellular and environmental cues: mitochondrial biogenesis (mitobiogenesis) and mitophagy (Liu et al. 2023).

Mitobiogenesis is characterized by continuous fusion and fission. Mitochondrial fusion neutralizes damaged mitochondrial components, whereas mitochondrial fission effectively separates damaged mitochondria from the entire mitochondrial architectural network for subsequent degradation by mitophagy (Chan 2020; Zhang et al. 2022). The balance between mitochondrial biogenesis and mitophagy regulates the control of mitochondrial quantity and quality.

**Fig.4:**
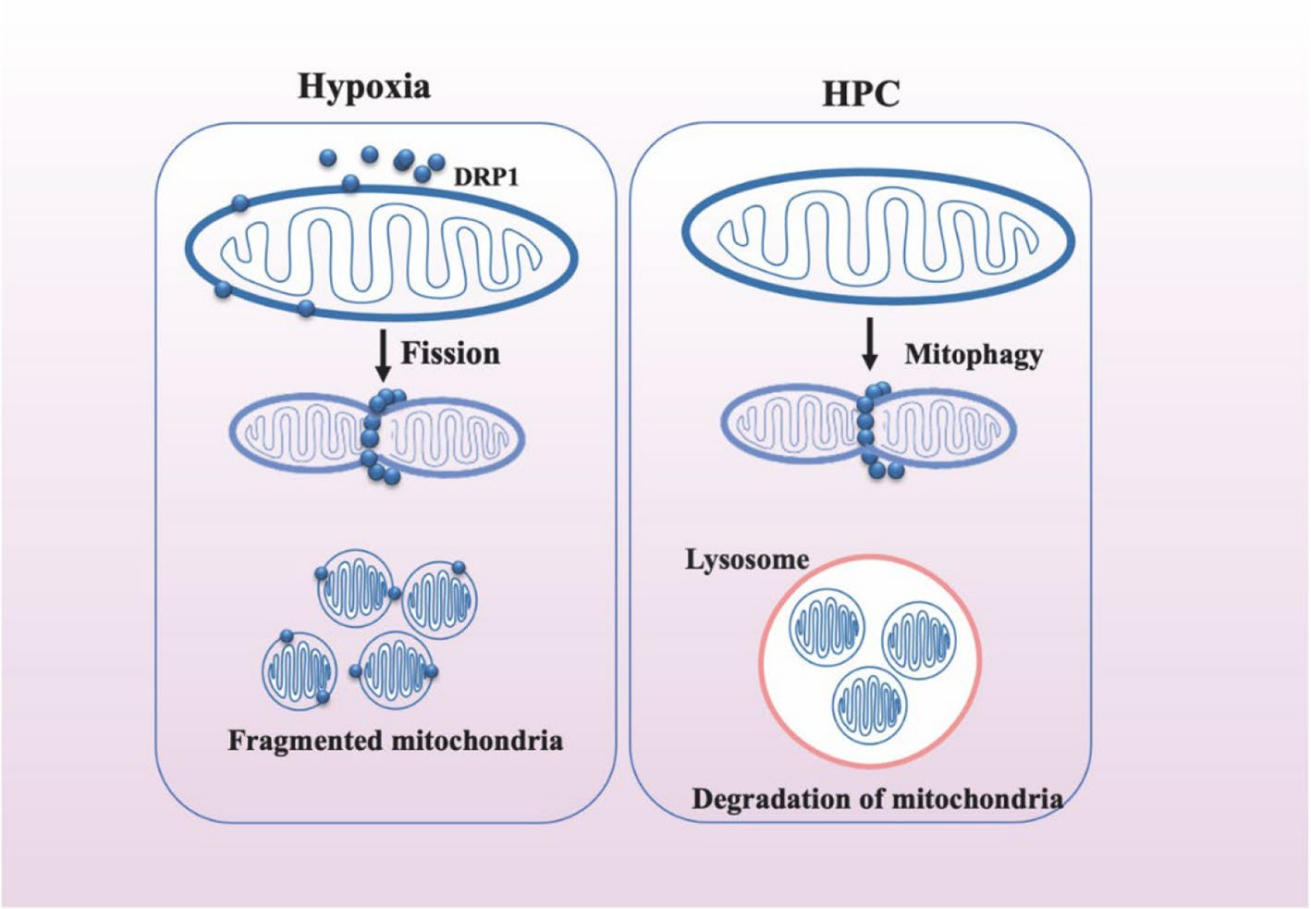
H/IPC inhibits mitochondrial fission. Abbreviations: Drp1, dynamin-related protein-1

Mitochondrial fusion is mediated by the fusion proteins mitofusin 1 and 2 (Mfn1/2) and optic atrophy 1 (OPA1), which are essential for the fusion of the outer and inner mitochondrial membranes, respectively. The fission process is executed by the dynamin family of GTPases, specifically dynamin-related protein-1 (Drp1) and mitochondrial fission protein 1 (Fis1) (Kraus et al. 2021). Drp1 induces mitochondrial fission when it is translocated from the cytosol to the outer mitochondrial membrane, leading to constriction and subsequent fission (Hao et al. 2023) (Fig. 4).

The intricate interplay among mitochondrial biogenesis, clearance, dynamics, and quality control systems forms an efficient defense mechanism against pathological stress, ensuring the maintenance of mitochondrial function. Zeng reported that activated Drp1 regulates p62-mediated autophagic flux and aggravates inflammation during cerebral ischemia/reperfusion (Zeng et al. 2022). Hypoxia impairs the balance of mitochondrial dynamics by increasing mitochondrial fragmentation (Han et al. 2022). Moreover, ischemia-induced cleavage of OPA1 at the S1 site aggravates mitochondrial fragmentation and reperfusion injury in neurons (Li et al. 2022). Mfn2 mediates cannabidiol-induced neuroprotection against cerebral ischemia in rats (Xu et al. 2023). Lu et al*.* reported that Mfn2 protects against cerebral reperfusion injury by regulating ROS levels and autophagic flux (Lu et al. 2025).

Dexmedetomidine enhances cell activity and decreases apoptosis to maintain blood–brain barrier integrity by inhibiting Drp1-related endothelial mitochondrial dysfunction during ischemic stroke (Zhou et al. 2021). The deletion of neuronal Drp1 improves mitochondrial respiratory capacity and Ca^2+^ homeostasis, attenuates superoxide production in response to ischemia and excitotoxicity in vitro and ex vivo, and reverses excessive stroke damage associated with mitochondrial fission (Flippo et al. 2020).

AMPK plays important roles in the molecular mechanism of mitochondrial biogenesis, fission, and fusion. AMPK is a major promoter of mitochondrial biogenesis, thus enhancing the ability of cells to provide energy substrates. AMPK controls mitochondrial biogenesis and synthesis of peroxisome PGC-1α mRNA in response to energy stress (Malik et al. 2023). C1q/tumor necrosis factor-related protein-3 promotes mitochondrial biogenesis and physiological functions in hippocampal neuronal cells after OGD via the AMPK/PGC-1α pathway (Gao et al. 2020). Fan et al*.* reported that heat shock protein 22 modulates mitochondrial biogenesis dependent on nuclear respiratory factor 1 (NRF1)/mitochondrial transcription factor A, as well as DRP1-induced mitochondrial apoptosis, through AMPK-PGC-1α signaling to alleviate early brain injury in rats with subarachnoid hemorrhage (Fan et al. 2021). Gao et al*.* reported that resveratrol attenuates cerebral ischemia‒reperfusion injury by activating the AMPK-Mfn1 pathway and that Mfn1 knockdown abolishes the beneficial effects of resveratrol on hypoxia-reoxygenation (HR)-treated N2a cells (Gao et al. 2019).

The neuroprotective effects of H/IPC are associated with mitochondrial biosynthesis, fusion, and fission. Sandhu et al*.* reported that IPC induces mitochondrial biogenesis in undifferentiated cell lines, resulting in an increase in mitochondrial mass and increased expression of genes necessary for mitochondrial biogenesis (nuclear factor erythroid 2-related factor1/2, Nrf1, and Nrf2) and function (cyclooxygenase-3). These changes translate to an increase in organelle copy numbers, which increase from an average of 20–40 mitochondria to 40–60 mitochondria per cell (Sandhu et al. 2022). In the transient middle cerebral artery occlusion (tMCAO)-IH group, as an effective measure of HPC, the AMPK-PGC-1α-Sirt3 signaling pathway was activated, mitochondrial biogenesis was promoted, mitochondrial ultrastructural damage was repaired, and mitochondrial function was improved, thereby reducing brain damage and promoting motor function recovery in rats with cerebral ischemia (Su et al. 2022). HPC improves mitochondrial function and reduces OGD-induced oxidative stress, thereby improving the structure and function of astrocytic mitochondria and promoting neuroprotection (Wu et al. 2021b).

At present, relatively minimal research on H/IPC related to mitochondrial biogenesis, fusion and fission in the nervous system has been conducted, and further studies are needed to elucidate its targets and to explore its molecular mechanisms.

### Other

In addition to the abovementioned aspects, H/IPC may provide protection by influencing mitochondrial DNA, the mitochondrial respiratory chain, the mitochondrial ATP-sensitive potassium channel, mPTP, and other factors. However, current research in these areas is limited, and further studies are needed to elucidate the underlying mechanisms and potential molecular pathways involved.

Directly targeting mitochondria, similar to their activation by H/IPC, may offer an effective approach to protect organs from hypoxia-induced ischemic injury. Current mitochondria-targeting drugs include adenosine and mitochondrial telomerases. Adenosine receptor A1 enhances mitochondrial biogenesis and exerts neuroprotective effects after cerebral ischemia through PGC-1α (Han et al. 2023). Ale-Agha et al*.* reported that mitochondrial telomerase reverse transcriptase protects against myocardial I/R injury by improving the composition and function of complex I (Ale-Agha et al. 2021). Artificial increases in synaptosomal SUMO1 and SENP1 levels modulate calcium intake and glutamate release (Feligioni et al. 2013). The selective mitochondrial potassium channel agonist diazoxide exhibits a protective effect against cerebral ischemia (O'Sullivan et al. 2007). Compared with other drugs, mitochondrial-targeting therapies can improve energy metabolism disorders and reduce apoptosis. Therefore, developing treatments that protect mitochondrial function holds important clinical significance.

## Conclusions and perspectives

Understanding the possible mechanisms of H/IPC is crucial for harnessing this endogenous cellular protective mechanism to treat hypoxia–ischemia-related diseases and protect individuals in extremely hypoxic environments from its harmful effects. Owing to the crucial role of mitochondrial metabolism in cerebral hypoxic–ischemic injury, how H/IPC maintains mitochondrial homeostasis by regulating mitochondrial quality is crucial for neuroprotection. Although some studies have shown that mitochondria are involved in H/IPC-mediated neuroprotection, the exact mechanism of this process remains unclear.

In addition, another main challenge currently faced by H/IPC is clinical trials. Some RIPC models have been developed for use in clinical trials. For example, Chen et al*.* investigated RIPC in 1776 patients with moderate ischemic stroke and reported an improvement in 90-day neurological outcomes if RIPC was initiated within 48 h of stroke onset and was performed in the bilateral arm twice daily for 14 days (Chen et al. 2022). Clinical translation of H/IPC is hampered by methodological challenges. For example, the optimal intensity, duration, and frequency of preconditioning stimuli remain uncertain, and interindividual variability in responsiveness has not been adequately addressed. Moreover, compliance issues and patient tolerance, particularly in elderly or critically ill populations, complicate the implementation of standardized protocols. Identifying robust molecular biomarkers to predict H/IPC responsiveness would greatly aid in patient stratification and personalized treatment planning (Table 1).

**Table 1:** Summary of mitochondrial mechanisms in H/IPC

Detection of relevant indicators	Effects produced by H/IPC	Related Regulators	Refs.
H/IPC and mitochondrial quality control	H/IPC regulates mitochondrial quality control and maintains mitochondrial homeostasis	Promote ATP production, downregulate ROS, activate autophagy, maintain Ca^2+^ transport, reduce mitochondrial fission, promote mitochondrial fusion, reduce apoptosis	Mitchell 1961; Neupane et al. 2019; Nunnari and Suomalainen 2012; Ren and Zhang 2018
H/IPC and ATP	H/IPC promotes mitochondrial ATP production	ATP production is regulated by MMP, PMF, AMPK, and Sirt3, stimulating glucose metabolism	Dingley et al. 2010; Shadel and Horvath 2015; Han et al. 2024; Hardie et al. 2012; Yan et al. 2025
H/IPC and ROS	ROS have a “double-edge” effect on stress, H/IPC downregulates ROS levels	reduced ROS levels; HIF-1α, AMPK-PGC1α and HIF-2α regulate ROS through cellular metabolism	Chen et al. 2023; Wu et al. 2021a; Li et al. 2019; Gardiner et al. 2022
H/IPC and Ca^2+^ overload	H/IPC inhibits Ca^2+^ influx to counter Ca^2+^ overload	Precise control of Ca^2+^ concentration through the modulation of calcium channels, mitochondrial Ca^2+^ uniporters and Na^+^-Ca^2+^exchangers and the H^+^/Ca^2+^ exchangers, upregulation of NCX1 and NCX3 activity reduces mitochondrial Ca^2+^ concentration	Filadi and Greotti 2021; Akyuva and Naziroglu 2020; Cerullo et al. 2018; Brancaccio et al. 2022; Sisalli et al. 2014
H/IPC and mitochondria-mediated intrinsic apoptosis pathway	HPC inhibits apoptosis	BCL-2 family of proteins (such as Bax, anti-apoptotic) activates Apaf-1 and the transforming growth factor/Smad2/3 pathway	Singh et al. 2019; Sun et al. 2021; Huang et al. 2019; Zhang et al. 2021b; Guan et al. 2019
H/IPC and mitophagy	Autophagy is involved in the protective effects of HPC	The ubiquitin-mediated pathway and the receptor-mediated pathway (such as PINK1, BNIP3, FUNDC1)	He et al. 2022; Shen et al. 2021; Wen et al. 2021
H/IPC and mitochondrial biogenesis, fission, fusion	H/IPC inhibits mitochondrial fission, promotes mitochondrial fusion, and maintains mitochondrial homeostasis	Mitochondrial fusion is mediated by the fusion proteins MFN1/2, OPA-1 and AMPK/PGC1; the fission process is executed by the dynamin family of GTPases (such as DRP1); AMPK controls mitochondrial biogenesis	Hao et al. 2023; Kraus et al. 2021; Malik et al. 2023; Sandhu et al. 2022; Su et al. 2022

H/IPC shows great promise as a metabolism-centered, noninvasive neuroprotective strategy. Current research on mitochondria, the primary focus of many investigators, has been conducted mainly using rodent models or in vitro systems. To identify potential clinical targets, further studies are needed. For mechanistic investigations, modern analytical methods can be employed, such as mitochondrial single-cell sequencing to detect mitochondrial DNA (mtDNA) variations, metabolic flux analysis to examine pathways, including glucose metabolism and the tricarboxylic acid cycle, and electron microscopy to assess mitochondrial ultrastructure.

## Data Availability

Not applicable.

## Abbreviations

H/IPC: Hypoxic–ischemic preconditioning
ATP: Adenosine triphosphate
IHH: Intermittent hypobaric hypoxia
OSAS: Obstructive sleep apnea syndrome
ETC: Electron transport chain
ROS: Reactive oxygen species
MMP: Mitochondrial membrane potential
NADH: Nicotinamide adenine dinucleotide
PMF: Proton motive force
RIPC: Remote ischemic preconditioning
mtON: Mitochondria-ON
AMPK: AMP-activated protein kinase
IH: Intermittent hypoxia
PGC-1α: Proliferator-activated receptor γ coactivator-1α
Sirts: Sirtuins
mtROS: Mitochondrial ROS
OGD: Oxygen glucose deprivation
CIRI: Cerebral ischemia‒reperfusion injury
HIF-1α: Hypoxia-inducible factor-1α
mPTP: Mitochondrial permeability transition pore
BCL-2: B-cell lymphoma 2
EPO: Erythropoietin
PINK1: PTEN-induced putative kinase protein 1
BNIP3: BCL2-interacting protein 3
FUNDC1: FUN14 domain-containing 1
HPPSC_EVs: Human placenta-derived mesenchymal stem cell-derived extracellular vesicles
Mfn1/2: Mitofusin 1 and 2
Drp1: Dynamin-related protein-1
tMCAO: Transient middle cerebral artery occlusion
